# Neurotoxicity of *Tityus bahiensis* (brown scorpion) venom in sympathetic vas deferens preparations and neuronal cells

**DOI:** 10.1007/s00204-020-02799-y

**Published:** 2020-06-16

**Authors:** Rita de Cássia Collaço, Stephen Hyslop, Thalita Rocha, Valquiria A. C. Dorce, Edward G. Rowan, Edson Antunes

**Affiliations:** 1 Department of Pharmacology, Faculty of Medical Sciences, State University of Campinas (UNICAMP), Campinas, SP Brazil; 2 Strathclyde Institute of Pharmacy and Biomedical Sciences, University of Strathclyde, Glasgow, UK; 3 São Francisco University (USF), Bragança Paulista, SP Brazil; 4 Laboratory of Pharmacology, Division for Scientific Development, Butantan Institute, São Paulo, SP Brazil

**Keywords:** Autonomic neurotransmission, Voltage-gated sodium channel, Tetrodotoxin, ND7-23 cells, Electrophysiology

## Abstract

Systemic scorpion envenomation is characterized by massive neurotransmitter release from peripheral nerves mediated primarily by scorpion venoms neurotoxins. *Tityus bahiensis* is one of the medically most important species in Brazil, but its venom pharmacology, especially regarding to peripheral nervous system, is poorly understood. Here, we evaluated the *T. bahiensis* venom activity on autonomic (sympathetic) neurotransmission by using a variety of approaches, including vas deferens twitch-tension recordings, electrophysiological measurements (resting membrane potentials, spontaneous excitatory junctional potentials and whole-cell patch-clamp), calcium imaging and histomorphological analysis. Low concentrations of venom (≤ 3 μg/mL) facilitated the electrically stimulated vas deferens contractions without affecting postsynaptic receptors or damaging the smooth muscle cells. Transient TTX-sensitive sustained contractions and resting membrane depolarization were mediated mainly by massive spontaneous ATP release. High venom concentrations (≥ 10 μg/mL) blocked the muscle contractions and induced membrane depolarization. In neuronal cells (ND7-23*wt*), the venom increased the peak sodium current, modified the current-voltage relationship by left-shifting the Na_v_-channel activation curve, thereby facilitating the opening of these channels. The venom also caused a time-dependent increase in neuronal calcium influx. These results indicate that the sympathetic hyperstimulation observed in systemic envenomation is presynaptically driven, probably through the interaction of α- and β-toxins with neuronal sodium channels.

## Article Highlights

- *Tityus bahiensis* scorpion venom modulates neurotransmission in mouse vas deferens
- Facilitation at low venom concentrations involves a presynaptic action
- The venom mobilizes intracellular calcium in neurons without causing tissue damage
- Sodium channel modulation by venom toxins accounts for most of these effects

## Introduction

Scorpion envenomation is a major public health problem, especially in subtropical and tropical regions of developing countries (Chippaux 2012). In Brazil, the number of registered cases has increased by 332% over the last 10 years–> 200,000 cases/year, and scorpionism is now considered the primary cause of envenomation by venomous animals with *T. bahiensis* (brown scorpion) and *T. serrulatus* (yellow scorpion) being the two most clinically important species (Brasil. Ministério da Saúde 2019; Bucaretchi et al. 2014).

Systemic envenomation occurs in less than a third of cases and can be life-threatening, especially in children, for whom the mortality rate is about 10 times higher than in adults (Albuquerque et al. 2013; Bucaretchi et al. 2014). The clinical manifestations of envenoming by *Tityus* spp. are mostly local, consisting of immediate severe pain and varying degrees of erythema, edema and paraesthesia (Bucaretchi et al. 2014; Cupo 2015). Systemic envenomation by *Tityus* spp. frequently results in an ‘autonomic storm’ that consists of a massive release of sympathetic and parasympathetic neurotransmitters that leads to a complex pattern of central and peripheral responses. Parasympathetic hyper-stimulation can trigger bradycardia, hypotension, myosis, bronchospasm, hypersalivation, profuse sweating, diarrhoea, vomiting and priapism, while sympathetic effects include tachycardia, hypertension, mydriasis, hyperglycemia, tremors and agitation (Chippaux 2012; Bucaretchi et al. 2014). The parasympathetic stimulation occurs immediately after the sting and tends to be less severe than the sympathetic stimulation, which is persistent and responsible for most lethal effects observed in the later stages of systemic envenomation (Bucaretchi et al. 2014; Isbister and Bawaskar 2014). Scorpion venoms also act on non-adrenergic non-cholinergic nitrergic nerve fibres causing relaxation of corpus cavernosum smooth muscle in vitro (Teixeira et al. 1998, 2003) and likely accounts for the reported priapism in children (Almitai et al. 1985; Santos et al. 2016). The “autonomic storm” on systemic scorpionism is commonly attributed to the action of neurotoxic peptides that modulate ion channel activity and, consequently, neurotransmitter release (Guieu et al. 1995; Lourenço et al. 2002; Pucca et al. 2015).

We have previously shown that *T. bahiensis* venom, at a low concentration similar to that found in clinical scorpionism, affects somatic neurotransmission by increasing evoked and spontaneous acetylcholine release, mainly through a presynaptic action, most likely mediated by α- and β-toxins interacting with axonal and nerve terminal tetrodotoxin (TTX)-sensitive sodium ion channels (Collaço et al. 2019). In the present work, we have extended our study of the neurotoxicity of *T. bahiensis* venom to examine its actions on the sympathetic neurotransmission, using the vas deferens smooth muscle preparation, which is highly innervated by sympathetic nerve fibres and can be bisected onto epidydimal (presenting phasic contraction predominantly purinergic) and prostatic (presenting tonic contraction predominantly noradrenergic) portions (Burnstock and Verkhratsky 2010), thus representing a classical model of sympathetic co-transmission (ATP and noradrenaline). Specifically, we used a combination of myographic, electrophysiological, calcium imaging and histopathological approaches in rat and mouse vas deferens to understand the mode of action of *T. bahiensis* venom on the autonomic nervous system.

## Material and methods

### Reagents and *T. bahiensis* venom

The reagents (analytical grade) for physiological solutions were purchased from J.T. Baker Chemicals/Mallinckrodt (Mexico City, DF, Mexico), Merck (Rio de Janeiro, RJ, Brazil) or local suppliers. Prazosin, pyridoxalphosphate-6-azophenyl-2′,4′-disulfonic acid (PPADS), adenosine 5′-triphosphate (ATP), noradrenaline (NA), tetrodotoxin (TTX) and dimethyl sulfoxide (DMSO) were obtained from Sigma-Aldrich Chemical Co. (St. Louis, MO, USA). The ND7-23 wild type cell line was purchased from Sigma-Aldrich (Irvine, Scotland, UK). *d*-Tubocurarine (dTc), the Fluo-4 AM and isoflurane were purchased from Abbott (Lake Bluff, IL, USA), Thermo Fisher Scientific Inc. (Waltham, MA, USA) and Cristália (Itapira, SP, Brazil), respectively. The media (Gibco^®^ RPMI 1640 and Gibco^®^ TrypLE™ Express) and solutions for cell culture were obtained from Life Technologies Ltd. (Paisley, Scotland, UK). Lyophilized *T. bahiensis* venom was provided by the Arthropods Laboratory of the Butantan Institute (São Paulo, Brazil) and was obtained from scorpions of both sexes. This work was registered with the Brazilian National System for the Management of Genetic Resources and Associated Traditional Knowledge (SisGen, protocol no. A5F1946).

### Animals

Male Balb/c mice (20–35 g, 8-week old) and male Wistar rats (300–400 g; 12-week old) were obtained from the Multidisciplinary Centre for Biological Investigation (CEMIB, the central animal house at UNICAMP) or from the Strathclyde Institute of Pharmacy and Biomedical Sciences. The animals were housed in plastic cages (mice and rats; 5/cage with a wood shaving substrate) at 22 ± 3 °C on a 12 h light/dark cycle with free access to standard rodent chow (Nuvital^®^) and water. The experiments were approved by an institutional Committee for Ethics in Animal Use (CEUA/UNICAMP, Protocols Nos. 4068-1 and 4189-1) and were done according to the general ethical guidelines of the Brazilian Society for Laboratory Animal Science (SBCAL), the Brazilian National Council for Animal Experimentation (CONCEA), Brazilian legislation (Federal Law no. 11794 of October 8, 2008), EU Directive 2010/63/EEC for the Protection of Animals Used for Scientific Purposes and the UK Government Animals (Scientific Procedures) Act (ASPA) 1986 and associated guidelines.

### Vas deferens preparation

Rats and mice were euthanized with an overdose of isoflurane (via inhalation) and the vas deferens on both sides were carefully removed and bisected into two portions: the epididymal end, which is predominantly purinergic, and the prostatic-end portion, which is predominantly noradrenergic (Burnstock and Verkhratsky 2010). The preparations were kept in Tyrode solution of the following composition (in mM): NaCl 137, KCl 2.7, CaCl_2_ 1.8, MgCl_2_ 0.49, NaH_2_PO_4_ 0.42, NaHCO_3_ 11.9 and glucose 11.1, constantly gassed with carbogen (95% O_2_ and 5% CO_2_) at 37 °C.

### Myographic studies

Prostatic and epididymal portions of rat was deferens were mounted under a resting tension of 1 g in a 5 mL organ baths containing Tyrode solution (as described previously). Muscle contractions were measured isometrically with a PanLab TRI201AD isometric transducer (Harvard Apparatus, Holliston, MA, USA) via a four-channel PowerLab ML866 physiograph coupled to a Quad Bridge ML224 amplifier and a computer containing LabChart7 software (all from ADInstruments, Newcastle, NSW, Australia).

#### Electrical-field stimulation (EFS)

The preparations were allowed to stabilize for 40 min before addition of *T. bahiensis* venom (1–30 μg/mL). Control preparations were incubated with Tyrode solution alone. A bipolar platinum ring electrode was placed around the muscle for field stimulation and connected to a Grass S48 stimulator (10 Hz, 5 ms, 60 V; Grass Medical Instruments, Quincy, MA, USA) using trains of stimuli of 1 s and 10 s duration for the prostatic and epididymal portions, respectively. The twitch-tension of both portions (phasic contractions of the prostatic portion and tonic contractions of the epididymal portion) were measured every 10 min and the results were expressed as a percentage (%) of the basal value before venom addition. At the end of the protocols, the vas deferens were fixed in 10% formaldehyde for histopathological analysis.

#### Pre-incubation with TTX or receptor antagonists

Separate preparations were subjected to EFS and the tissue was incubated with either TTX (200 nM; to abolish neurogenic activity) or the receptor antagonists prazosin (α_1_-adrenergic receptor antagonist, 1 μM), PPADS (purinergic P2X receptor antagonist, 30 μM) or *d*-tubocurarine (*d*-Tc, nicotinic receptor antagonist, 82 μM). After stabilization, the electrical stimulus was discontinued and the *T. bahiensis* venom (10 μg/mL; concentration chosen based on the myographic experiments) was added to the preparations.

#### Concentration–response curves to ATP and noradrenaline

The preparations were allowed to stabilize for 40 min before starting the concentration–response curves to ATP (100 nM–10 mM; prostatic end) and noradrenaline (3 nM–300 µM; epididymal end) in control preparations (incubated with Tyrode solution alone) and after 30 min incubation with *T. bahiensis* venom (10 μg/mL; same concentration as used in antagonist pre-incubation experiments). These experiments were also performed after the tissue was incubated with TTX (200 nM) for 30 min. Some experiments were also done in tissues pre-incubated with 200 nM TTX for 30 min.

### Histomorphological analysis

After the functional assays, the tissue samples were fixed in formaldehyde (10%) for further paraffin embedding processing. The tissues were then dehydrated in an ascending series of ethanol (70, 80, 90 and 100% for 30 min at each concentration), diaphanized in ethanol:xylol (1:1; 30 min), cleared in 100% xylol (30 min), and embedded in paraffin:xylol (1:1; 30 min) followed by pure paraffin (180 min) (PT-09TS, DP-2010, PR-10D, PA-2012; Lupetec, São Carlos, SP, Brazil). The paraffin blocks were sectioned (5 µm) using an MRP-03 Lupetec microtome and the sections were collected onto silanized glass slides. The histological sections were then deparaffinized in xylol, hydrated in a descending series of ethanol (100–70%) and deionized water for 5 min for each bath. Some slides were stained with hematoxylin–eosin (HE) while others were stained with Masson’s trichrome (MT) prior to morphometric analysis. Finally, the sections were dehydrated in an ascending ethanol series and xylol and mounted in Synthetic Canada balsam for further analysis with an Eclipse E200 Nikon microscope (Melville, NY, USA) using the NIS software (Elements AR4.60.00.64; Nikon, Tokyo, Japan).

### Electrophysiological studies

#### Resting membrane potentials and Spontaneous Excitatory Junctional Potentials

The prostatic portion of mouse vas deferens was opened longitudinally (the luminal mucosa was carefully removed) and pinned to a Sylgard-based (Dow-Corning, Allesley, Coventry, UK) perfusion chamber containing Tyrode solution connected to a temperature controller (Hugo Sachs Elektronic, Munich, Germany). For resting membrane potential measurements, borosilicate glass microelectrodes (GC120F; Harvard Apparatus Ltd., Kent, UK) produced with a model P-30 vertical puller (Sutter Instruments, Novato, CA, USA) and filled with 3 M KCl (resistance 60–90 MΩ) were inserted superficially into smooth muscle cells using a Leica Letz mechanical manipulator. The potential difference between the reference electrode (AgAgCl pellet) and the recording microelectrode was measured with a unitary gain high input impedance electrometer (model Electro 705, World Precision Instruments, Hertfordshire, UK). The membrane potentials were amplified using a CED 1902 (Cambridge Electronic Design, Cambridge, UK) and digitized with a NIQAQ-MX 16bit A/D convertor connected via a BCN-2110 connector block (National Instruments, Newbury, UK), and the potentials were recorded and analysed using WinEDR software (John Dempster, University of Strathclyde, UK). Experiments were carried out only if the following criteria were satisfied: (i) an abrupt penetration in the cell and immediate change in a potential to a more negative value than the initial, (ii) the membrane potential was stable and (iii) the impalement was stable for at least 5 min.

The resting membrane potential (RMP) and the spontaneous excitatory junctional potentials (SEJP) were measured before and after incubation with venom (0.3 μg/mL at 5, 10, 15, 30, 60, 90 and 120 min). The venom concentration was chosen based on preliminary studies in which (i) concentrations of 1 and 3 μg/mL promoted intense fasciculation in the tissue interfering with the recording by pulling the microelectrode out of the cells, and (ii) higher concentrations (10 and 30 μg/mL) induced a marked and long-lasting RMP depolarization and blocked the excitatory potentials recording.

### Neuronal cell culture

The ND7-23 wild type (ND7-23*wt*) neuronal cells were cultured in 25 cm^2^ flasks in Gibco^®^ RPMI 1640 medium supplemented with fetal bovine serum (10%), streptomycin (1%), penicillin (1%), L-glutamine (1%), sodium pyruvate (1%) and non-essential amino acids (1%) at 37 °C in a humidified atmosphere containing 5% CO_⁠2_. The culture media was changed at least every 3 days or as required. Cell dissociation was achieved by adding Gibco^®^ TrypLE™ Express for 3 min followed by centrifugation (1000 rpm, 2.5 min) and resuspension in Gibco^®^ RPMI 1640 media prior to plating on 13 mm coverslips for calcium imaging or suspension in external solution for patch-clamp assays.

#### Whole cell patch-clamp

For sodium channel recordings, the cells were maintained in external solution (composition, in mM: NaCl 129, KCl 3.25, CaCl_2_ 2, MgCl_2_ 2, HEPES 10, D-glucose 11, TEA-Cl 20, pH 7.2–7.4 adjusted with NaOH) and voltage-clamped at room temperature (20–22 °C) in the whole-cell configuration mode using an Axopatch 1D (Axon Instruments, Molecular Devices, Sunnyvale, CA, USA) controlled by Whole Cell Analysis Program (WCP) v.4.2.0 (John Dempster, University of Strathclyde) running on a Dell computer that was used for data acquisition and pulse generation. The data were filtered at 2 kHz and sampled at 10 kHz. For the whole-cell patch clamp, fire-polished patch pipettes were made from borosilicate glass capillaries (GC150F-10, Harvard Apparatus Ltd., Kent, UK) using a Sutter P-87 puller (Sutter Instruments Co., Novato, CA, USA) and had a resistance of 1.5–3 MΩ when filled with internal pipette solution (composition in mM: CsF 120, NaCl 10, HEPES 10, EGTA 11, TEA-Cl 10, CaCl_2_ 1, MgCl_2_ 1, pH 7.2–7.4 adjusted with KOH). Capacitative transients were electronically cancelled and voltage errors were minimised by applying between 75–95% series resistance compensation. Linear leak currents were subtracted off-line using a P/4 subtraction protocol. Currents were scrutinized for voltage artefacts and the current–voltage relationships obtained were characteristic of appropriately clamped cells. Total sodium currents were evoked by a 25 ms voltage step from the holding potential (− 120 mV) to a command potential (− 90 to + 80 mV) in 5 mV steps every second and recorded before and 10 min after the addition of *T. bahiensis* venom (10 µg/mL). The concentration was chosen based on preliminary studies in which (i) concentrations of 1 and 3 μg/mL takes longer to start acting than the cells can remain satisfactorily clamped, and (ii) higher concentration (30 μg/mL) blocked the sodium current.

#### Calcium imaging

Intracellular Ca^2+^ transients were monitored in ND7-23 wild type neurons using the Ca^2+^ sensitive fluorescent indicator Fluo-4 AM (Biotium Inc., Hayward, CA, USA) prepared as a 1 mM stock solution in dimethyl sulfoxide (DMSO; Sigma-Aldrich) and stored at − 20 °C.

Coverslips with ND7-23 cells were incubated with 4 μM Fluo-4 (diluted in culture medium) for 1 h in a tissue culture incubator. Before recording the Ca^2+^ transients, the coverslips were washed with Tyrode solution to remove extracellular indicator and left to equilibrate in the bath solution for 20 min before the addition of venom (0.3 µg/mL; concentration used in vas deferens electrophysiological experiments). All Ca^2+^ measurements were carried out at room temperature. The images were recorded using a Grasshopper3 USB3 camera model GS3-U3-15S5M-C (FLIR Integrated Imaging Solutions Inc., Richmond, Canada) attached to a Zeiss Axioskop 50 upright epifluorescence microscope (Carl Zeiss, Oberkochen, Germany). Excitation light was provided by a 50 W mercury short arc lamp (Osram, Germany) and filter set 9 (Carl Zeiss) that consisted of an excitation filter (BP 450–490 nm), beam splitter (FT 510 nm) and emission filter (LP 520 nm). Image acquisition was controlled with the software WinFluor software (John Dempster, University of Strathclyde, UK) and obtained at a rate of 2 frames/s and exposure times of 100–500 ms.

#### Statistical analysis

All results were expressed as the mean ± SEM, as indicated in the legends. Statistical comparisons were done using either Student’s unpaired t-test or two-way analysis of variance (ANOVA) followed by the Tukey–Kramer test, depending on the analysis, with *P* < 0.05 indicating significance. All data analyses were carried out using Prism v.6 (GraphPad Inc., La Jolla, CA, USA).

## Results

### Effects of *T. bahiensis* venom on EFS-induced rat vas deferens contractions

**Fig. 1:**
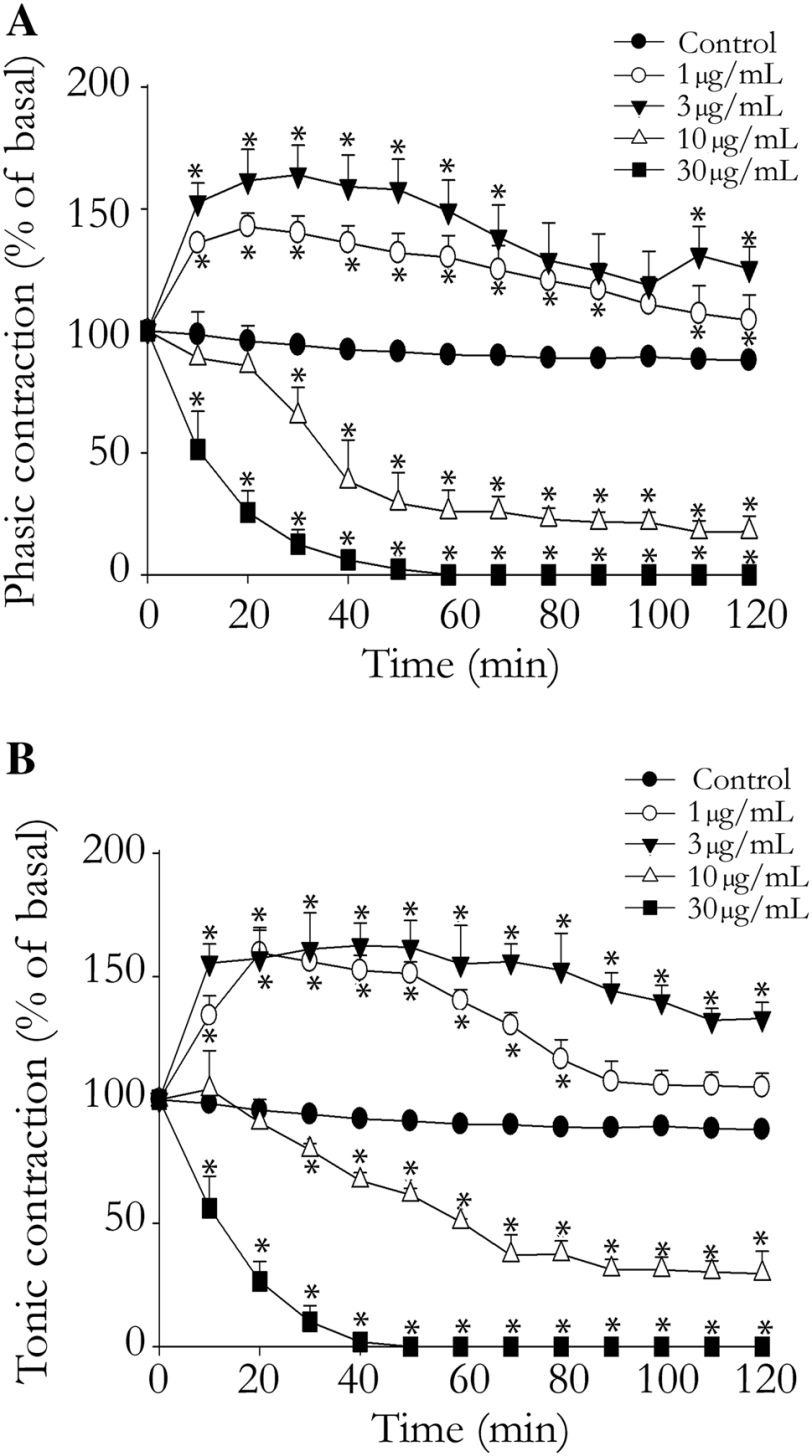
Sympathetic activity of *T. bahiensis* venom in field-stimulated bisected rat vas deferens. The preparations were field stimulated (10 Hz, 5 ms, 60 V; trains of 1 s for the prostatic portion and 10 s for the epididymal portion were applied every 2 min) and the phasic contractions of the prostatic portion (**a**); response predominantly purinergic) and tonic contractions of the epididymal portion (**b**); response predominantly noradrenergic) were recorded in the absence (control) and presence of *T. bahiensis* venom (1–30 µg/mL) for 120 min at 37 ºC. Note that low venom concentrations (1 and 3 µg/mL) caused muscle facilitation while higher concentrations (≥ 10 µg/mL) showed total blockade. The points represent the mean ± SEM of 4–6 experiments. *P < 0.05 compared to corresponding control responses

**Fig. 2:**
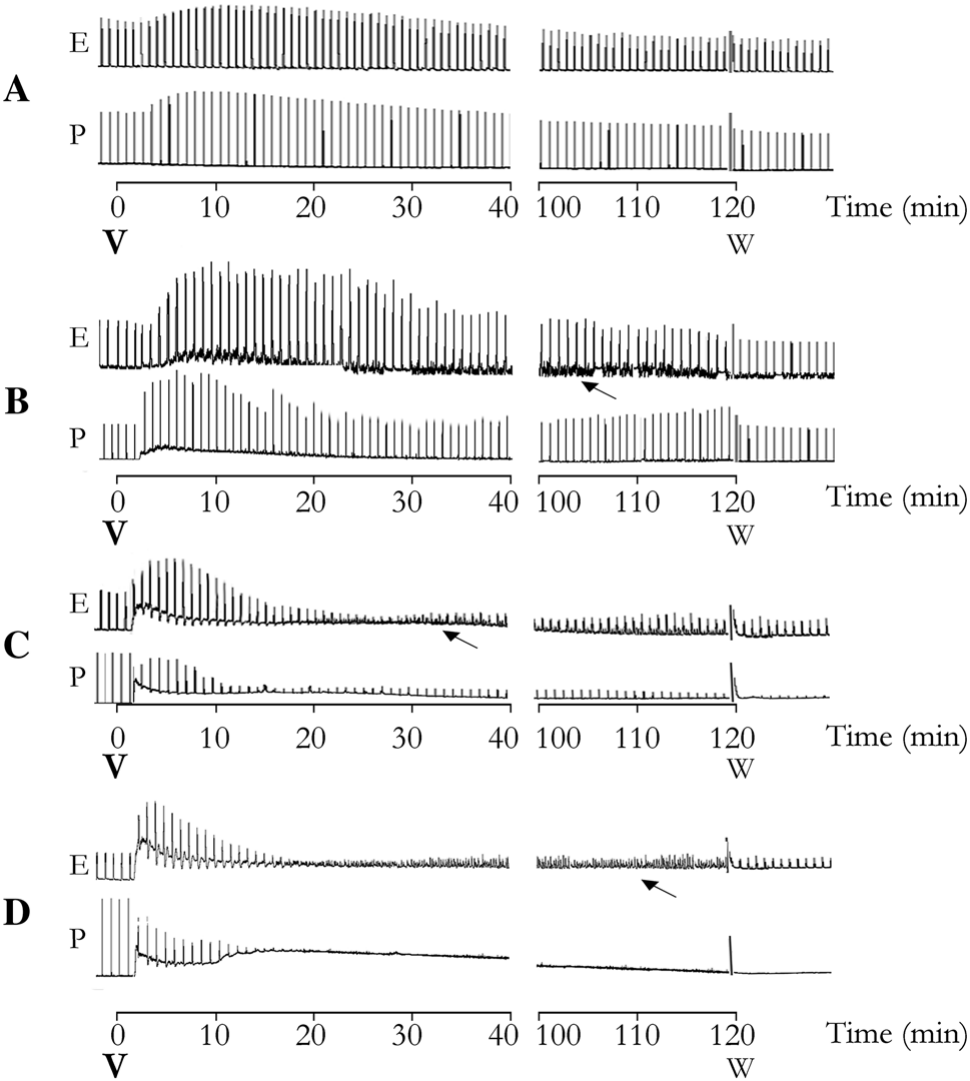
Representative recordings of the contractile responses of field-stimulated bisected rat vas deferens incubated with *T. bahiensis* venom. The prostatic (*P*) and epididymal (*E*) portions were field stimulated (10 Hz, 5 ms, 60 V; trains of 1 s for the prostatic portion and 10 s for the epididymal portion were applied every 2 min and incubated with venom (**a** − 1 µg/mL, **b** − 3 µg/mL, **c** − 10 µg/mL or **d** − 30 µg/mL) added at time 0. Note the sustained muscle contracture (increase in baseline tension) and fast spontaneous contractions/fasciculations (intermittent spikes) (↑) caused by the venom (≥ 3 µg/mL) in the epididymal portion. These recordings are representative of the mean values shown in (Fig. 1). *v * venom, *W* wash

The rat vas deferens provides a good model for investigating sympathetic cotransmission since the prostatic portion, which has a predominantly purinergic response, and the epididymal portion, which has a predominantly noradrenergic response, can be easily separated and studied independently. Prostatic and epididymal portions of the rat vas deferens preparations subjected to EFS were exposed to venom (1–30 μg/mL, 120 min). Low concentrations (1–3 μg/mL) of *T. bahiensis* venom caused marked facilitation of EFS-induced muscle contractions in both the prostatic and epididymal portions (Fig. 1a, b; Fig. 2a, b). A venom concentration of 10 μg/mL (Fig. 1a, b; Fig. 2c) produced a brief facilitation at 5-min (not shown) followed by a partial blockade in both portions (80 ± 8.5% and 88 ± 4.6% blockade at 120 min in the prostatic and epididymal portions, respectively) with the time to achieve 50% neuromuscular blockade being 45 ± 11 min and 31 ± 6 min (prostatic and epididymal portions, respectively). At 30 µg/mL, the EFS-induced contractions in both portions were totally blocked after 50–60 min (Fig. 1a, b; Fig. 2d). The venom also caused sustained muscle contraction (seen as an increase in baseline tension) in both portions after incubation with ≥ 3 µg/mL, and small spontaneous contractions (intermittent spikes) were also observed, especially in the epididymal portion (Fig. 2).

**Fig. 3:**
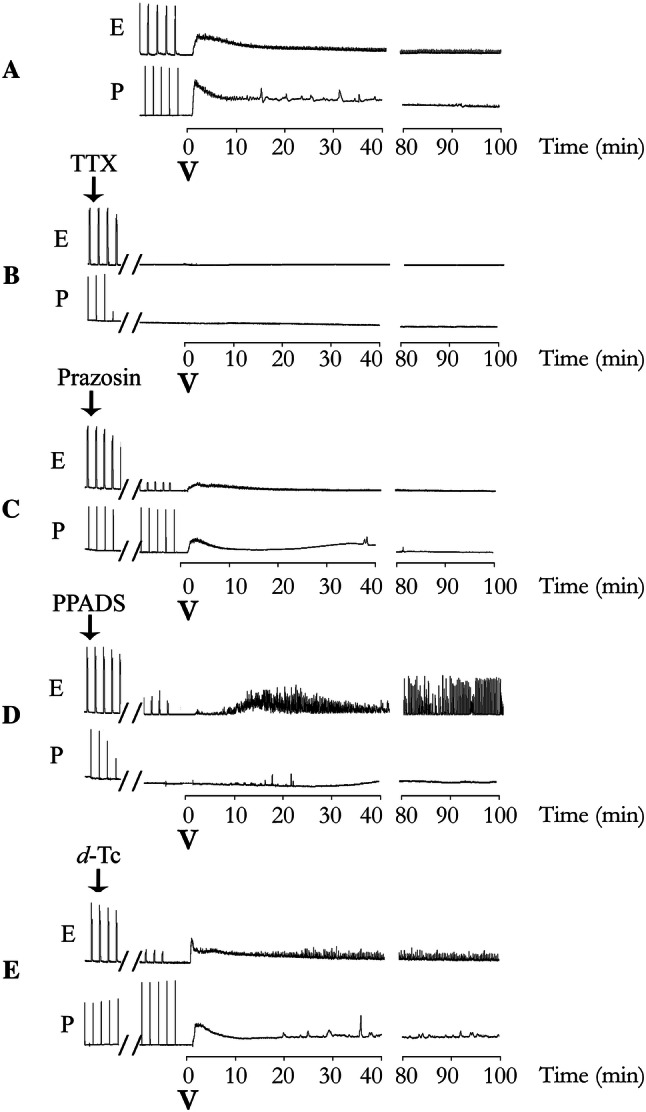
Contractile activity of field-stimulated bisected rat vas deferens incubated with *T. bahiensis* venom in the presence of tetrodotoxin (TTX) or different receptor antagonists. The prostatic (P) and epididymal (E) portions were field stimulated (10 Hz, 5 ms, 60 V; trains of 1 s for the prostatic portion and 10 s for the epididymal portion were applied every 2 min and the preparations were preincubated in Tyrode solution in the absence (**a**; control) or presence of TTX (**b** 200 nM), prazosin (**c** 1 µM), PPADS (**d** 30 µM) or *d*-tubocurarine (**e***d*-Tc; 82 µM). After the effects of the antagonists on muscle contraction were stabilized, the electrical stimulation was turned off and venom (V; 10 µg/mL) was added. Note that (**i**) the venom caused a sustained contraction even in the absence of electrical field stimulation (panel **a**), (**ii**) TTX and PPADS inhibited the venom-induced increase in baseline tension (prolonged muscle contraction), (**iii**) prazosin only partially reduced the increase in baseline tension but prevented the small fast contractions, and (**iv**) *d*-Tc did not affect the venom-induced increase in baseline tension or the small fast contractions (intermittent spikes). The recordings are representative of the mean values of 5–7 experiments for each condition shown

The origin of the sustained contraction was pharmacologically studied by testing *T. bahiensis* venom in the vas deferens preparations in the absence of electrical stimulation. Venom (10 µg/mL), added to both portions of vas deferens and myographic recorded for 100 min, still promoting the sustained muscle contraction that, apparently, did not depend on electrically induced nerve action potentials since the phenomenon persisted in the absence of electrical stimulation (Fig. 3a). However, the presence of functional Nav channels (on the sympathetic nerves) was required since the contracture was abolished by pre-treating the tissue with 200 nM TTX (used to block neuronal sodium channels) (Fig. 3b) suggesting a prejunctional site of action.

Preincubation with prazosin (α_1_-adrenergic receptor antagonist) caused progressive blockade of the EFS-induced responses in the epididymal portion of the vas deferens, but not in the prostatic portion (Fig. 3c), which agrees with the predominantly noradrenergic neurotransmission in this portion. The venom-induced contraction was partly reduced by prazosin (55 ± 3% reduction in the prostatic portion and 67 ± 1% in the epididymal portion), indicating a role for NA mediating this response. The small spontaneous contractions (intermittent spikes), were inhibited by prazosin in both portions of the vas deferens (Fig. 3c).

PPADS, a purinergic P2X antagonist, caused rapid and complete blockade of EFS-induced induced contractions in the prostatic (but not the epididymal) portion of the vas deferens that agrees with the predominantly purinergic neurotransmission in this portion (Fig. 3d). The immediate venom-induced contractions were totally inhibited by PPADS in both portions, confirming a major role for ATP in this response (Fig. 3d). Unexpectedly, in the epididymal portion, this antagonist markedly increased the frequency and amplitude of the intermittent spikes (Fig. 3d).

Preincubation with *d*-Tc did not effectively reduce the venom-induced increase in baseline tension or intermittent spikes in both portions (7 ± 4% reduction to epididymal portion and 25 ± 4% to prostatic portion) (Fig. 3e), indicating that presynaptic nicotine receptors do not have a crucial role in this response.

#### Effects of *T. bahiensis* venom on ATP- and noradrenaline-induced vas deferens contractions

**Fig. 4:**
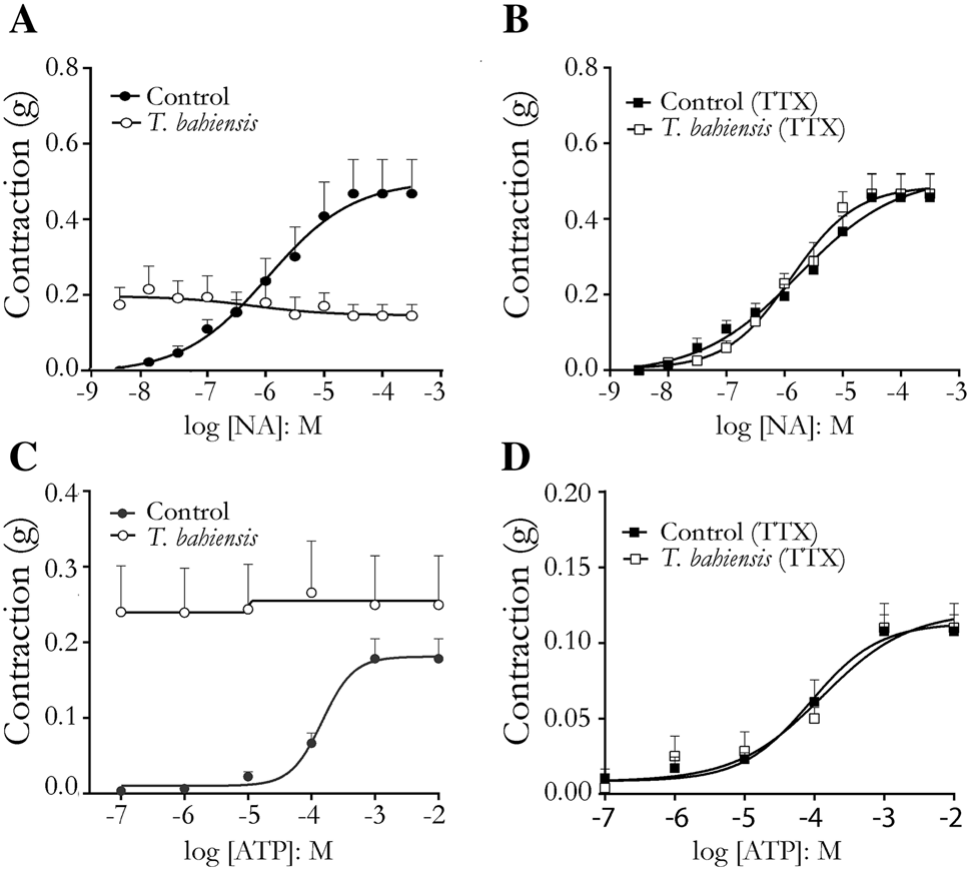
Effect of *T. bahiensis* venom (10 µg/mL) on concentration–response curves to exogenous noradrenaline (NA) and ATP in rat bisected vas deferens. Venom alone (V) caused a sustained contraction in the epididymal (**a**) and prostatic (**c**) portions and, even after a 60 min incubation, interfered with the normal concentration–response curve to exogenous NA and ATP. In tissues preincubated with 200 nM tetrodotoxin (TTX, added 30 min before venom addition), the venom did not affect the concentration–response curves to NA in the epididymal portion (**b**) or ATP in the prostatic portion (**d**) when compared to the responses in tissues without venom. The points represent the mean ± SEM of 4–7 experiments

To assess the interaction of venom toxins with postsynaptic receptors as a possible mechanism involved in the facilitation and blockade of contractile responses, the increase in baseline tension and the occurrence of intermittent spikes, we generated cumulative concentration–response curves to exogenous noradrenaline (NA) or ATP in the absence or presence of TTX (used to exclude prejunctional activity). Addition of NA (3–300 µM to the epididymal end) produced concentration-dependent contractions with potency (pD_2_) and Emax values of − 5.75 ± 0.17 and 0.51 ± 0.041, respectively (Fig. 4a, b). Addition of ATP (100 nM–10 mM to the prostatic end) produced concentration-dependent contractions with potency (pD_2_) and Emax values of − 3.83 ± 0.12 and 0.18 ± 0.01, respectively (Fig. 4c, d). The NA-induced contractile responses were not affected by TTX (200 nM); however, TTX significantly (*P* < 0.05) reduced the ATP-induced contractions to 0.11 ± 0.008 (Emax) without significantly affecting the pD_2_ (− 3.89 ± 0.02), confirming previous studies showing that TTX affects the exogenous ATP-induced contractions (Tam et al. 1997).

*T. bahiensis* venom (10 µg/mL) alone caused an immediate sustained contraction in the epididymal and prostatic portions. After 60 min incubation with venom, concentration–response curves to NA (3 nM–300 µM; epididymal portion) or ATP (100 nM–10 mM; prostatic portion) were performed. Under these experimental conditions, as the vas deferens smooth muscles still presenting the venom-induced contraction, the cumulative addition of cumulative NA or ATP did not affect the smooth muscle tone at any concentration used (Fig. 4a, c, respectively). On the other hand, in tissues pre-treated with TTX to abolish possible prejunctional actions of the venom, concentration–response curves to NA and ATP (pD_2_ of 0.49 ± 0.017 and Emax of − 0.89 ± 0.083 for NA; and pD_2_ of 0.12 ± 0.21 and Emax of − 4.06 ± 0.008 for ATP) were not different from those to these agonists in the absence of venom (Fig. 4b, d). This result strongly suggests that the site of action of the venom is not mediated via postsynaptic alpha-adrenoceptors or P2X receptors.

### Histopathological analysis

**Fig. 5:**
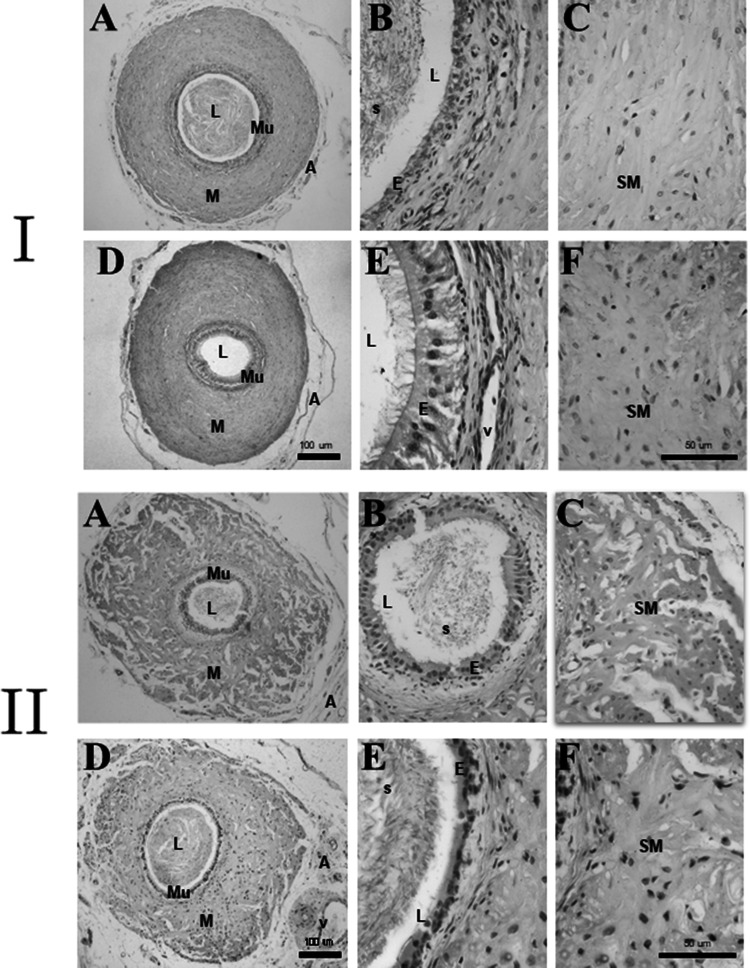
Histological analysis of the prostatic (I) and epididymal (II) portions of rat vas deferens after incubation with *T. bahiensis* venom (10 μg/mL) for 120 min. In each set of panels (I and II), the tissues were incubated with Tyrode solution alone (**a**–**c**; control muscles) or venom (**d**–**f**) for 120 min and then processed and analysed after staining with HE. Note the well preserved histomorphology of the mucosa (*Mu*) with pseudostratified columnar epithelium containing stereocilia (*E*) and the muscle layer (*M*) composed of smooth muscle (*SM*) and adventitia (*A*) with connective tissue. *L*  lumen; * s* spermatozoids; *v*  vessels. Scale bars: Panel I and II – **a** and **d** = 100 μm;** b**,** c**,** e** and** f** = 50 μm

Histopathological analysis of the epididymal and prostatic portions of rat vas deferens after the pharmacological protocols showed that *T. bahiensis* venom did not cause any major tissue damage (Fig. 5). Control and venom-treated tissues showed similar normal morphology with spermatozoids in the lumen, mucosa with a normal pseudostratified columnar epithelium and stereocilia, the presence of underlying connective tissue, normal smooth muscle fibres in the inner and outer longitudinal layers and middle circular layer, blood vessels and nerves. There was no ulceration, denudation, inflammation or myotoxicity. These results indicated that tissue damage was not a contributing factor to the responses of vas deferens tissue to *T. bahiensis* venom.

### Electrophysiological measurements

#### Vas deferens

The venom-induced increase in baseline tension in the prostatic portion of the vas deferens suggested the occurrence of spontaneous presynaptic activity that was mediated predominantly by ATP (based on the results shown in Fig. 3d). In view of this, we used conventional microelectrode techniques to examine the spontaneous release of ATP release in this tissue in the absence and presence of venom. Mouse vas deferens smooth muscle cells had a resting membrane potential (RMP) that varied from − 101 mV to − 72 mV during the 90 min experiment. Incubation with venom (0.3 µg/mL) depolarized the RMP (from 69.8 ± 1.96 mV (basal, control) to − 51 ± 1.7 mV (*P* < 0.05), − 48.2 ± 11 mV (*P* < 0.05), − 62.8 ± 11 mV (*P* < 0.05), − 72.7 ± 14 mV (*P* < 0.05) and − 80.7 ± 7.2 mV after 5, 15, 30, 60 and 90 min of incubation, respectively (*n* = 8).

**Fig. 6:**
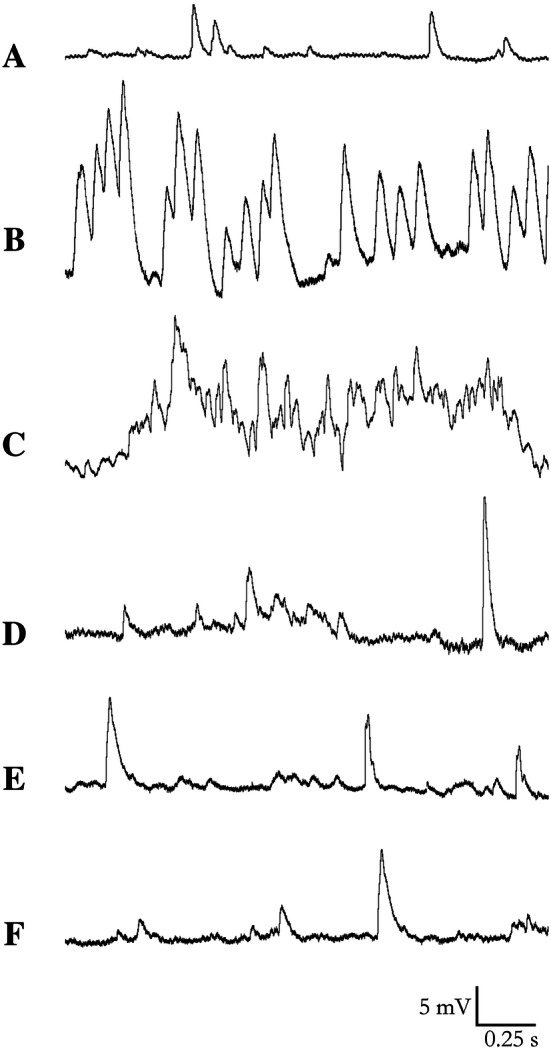
Representative recordings of spontaneous excitatory junction potentials (SEJP) in mouse vas deferens incubated without or with *T. bahiensis* venom (0.3 µg/mL). The SEJP were recorded from smooth muscle in the prostatic portion of the vas deferens by using conventional intracellular microelectrodes as described in the Methods before (**a**; basal) and 5 (**b**), 15 (**c**), 30 (**d**), 60 (**e**) and 90 min (**f**) after venom addition. Incubation with venom markedly increased the frequency and amplitude of SEJP in the first 15 min of incubation, followed by a gradual decrease in activity. The recordings are representative of the mean values of 5–6 experiments

Control spontaneous excitatory junction potentials (SEJP) had amplitudes mostly of 1.5–2.5 mV, although larger potentials (> 15 mV) were also occasionally observed (data not shown). Incubation with venom (0.3 µg/mL) caused a massive increase in SEJP amplitude and frequency that made it impossible to quantify the potentials (manually or by software) (Fig. 6). It is worth noting that the time frames for the changes in SEJP frequency and amplitude and RMP depolarization coincided with the increase in baseline tension (sustained contraction) seen in the myographical assays. Higher venom concentrations (≥ 10 µg/mL) resulted in dramatic RPM depolarization and the blockade of SEJP (data not shown).

#### Whole cell patch-clamp

**Fig. 7:**
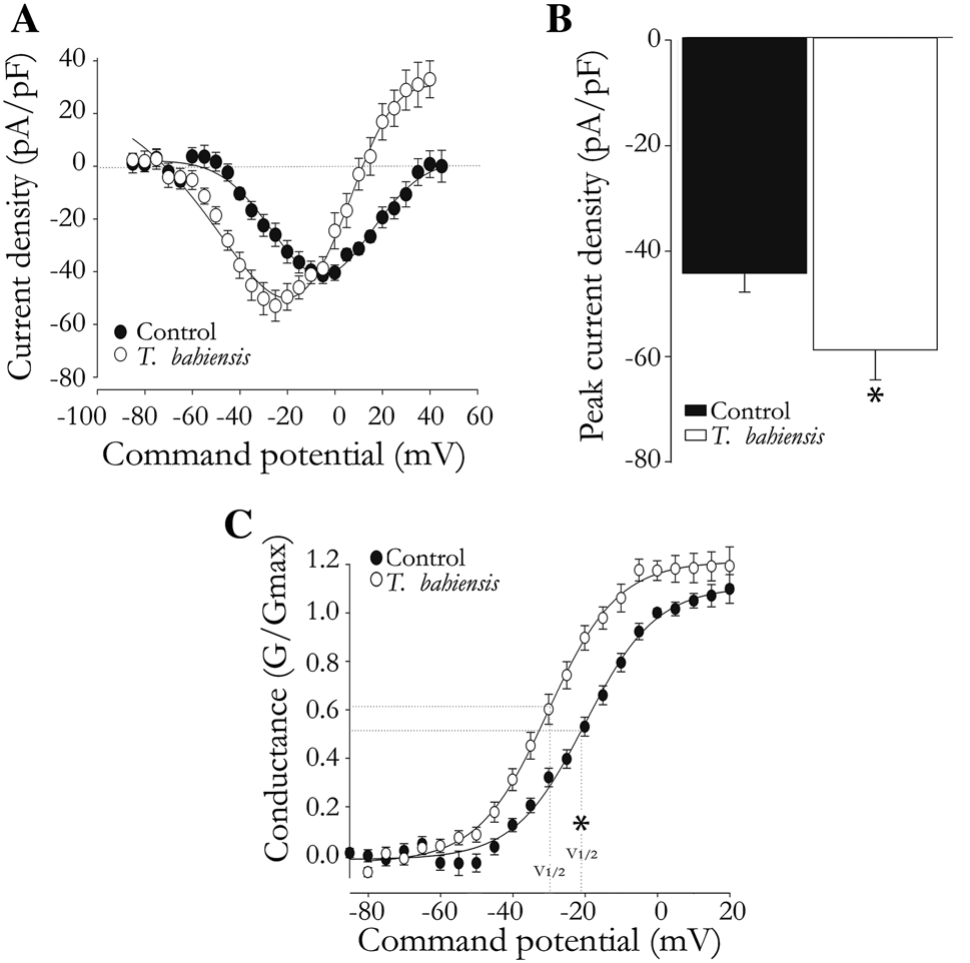
Effect of *T. bahiensis* venom (10 µg/mL) on sodium currents in ND7-23*wt* neurons. Incubation with venom shifted the current density versus command potential curve (I–V curve) to more negative potentials (**a**) and increased the sodium peak current density (**b**). **c** Boltzmann curves of average normalized conductance versus the command potential for control cells (slope 9.0  ±  0.72; *n* = 15) and after incubation with venom (slope 9.7  ±  0.43; *n* = 18). Note the venom-induced leftward shift in the activation curve. The points and columns represent the mean ± SEM of 15–18 experiments. *P < 0.05 compared to control cells

Since the results described so far indicated that *T. bahiensis* venom acted on vas deferens mainly via presynaptic, TTX-sensitive mechanisms, we examined the effect of the venom on sodium currents mediated by voltage-gated sodium Na_v_ 1.7, as this sodium channel subtype is responsible for 65% of the peak inward current, followed by Nav1.6 (∼20%) in ND7-23 *wt* cells (Rogers et al. 2016). The neuronal membrane potential was held at − 120 mV to ensure that steady-state sodium channel inactivation was negligible and that the maximum number of sodium channels was available during the pulse protocol. The latter consisted of a series of 5 mV depolarization steps from − 85 mV (holding potential) to  + 40–45 mV (command voltages). In control cells and in basal recordings in venom-treated cells, the inward currents initiated when the command potential was greater than − 35 mV, peaked around − 5 mV and reversed direction at + 44.6 ± 2.31 mV, as shown by the voltage-current (*I-V*) curve (Fig. 7a). After incubation with *T. bahiensis* venom (10 µg/mL), the *I-V* curve was modified and the currents initiated at − 55 mV, peaked around − 25 mV and reversed at + 20  ±  2.8 mV. Incubation with venom also increased the density of the peak current compared to control recordings (Fig. 7b).

The venom also modified the sodium channel conductance, assessed as the voltage at which half maximum (V_1/2_) activation occurred; this value was significantly lower (more negative voltage) in tissues incubated with venom than in control tissues (venom-treated: − 30 ± 0.46 mV vs. control: − 21 ± 0.79 mV, *n* = 15–18; *P* < 0.05), although the slope of the activation curve was unaltered. In addition to the V_1/2_, the activation curve was also shifted to the left after incubation with venom, indicating that the voltage-dependent activation had moved to more hyperpolarized potentials (Fig. 7c). Overall, these results suggested that channel activation was facilitated by the venom.

### Intracellular Ca^2+^ mobilization

**Fig. 8:**
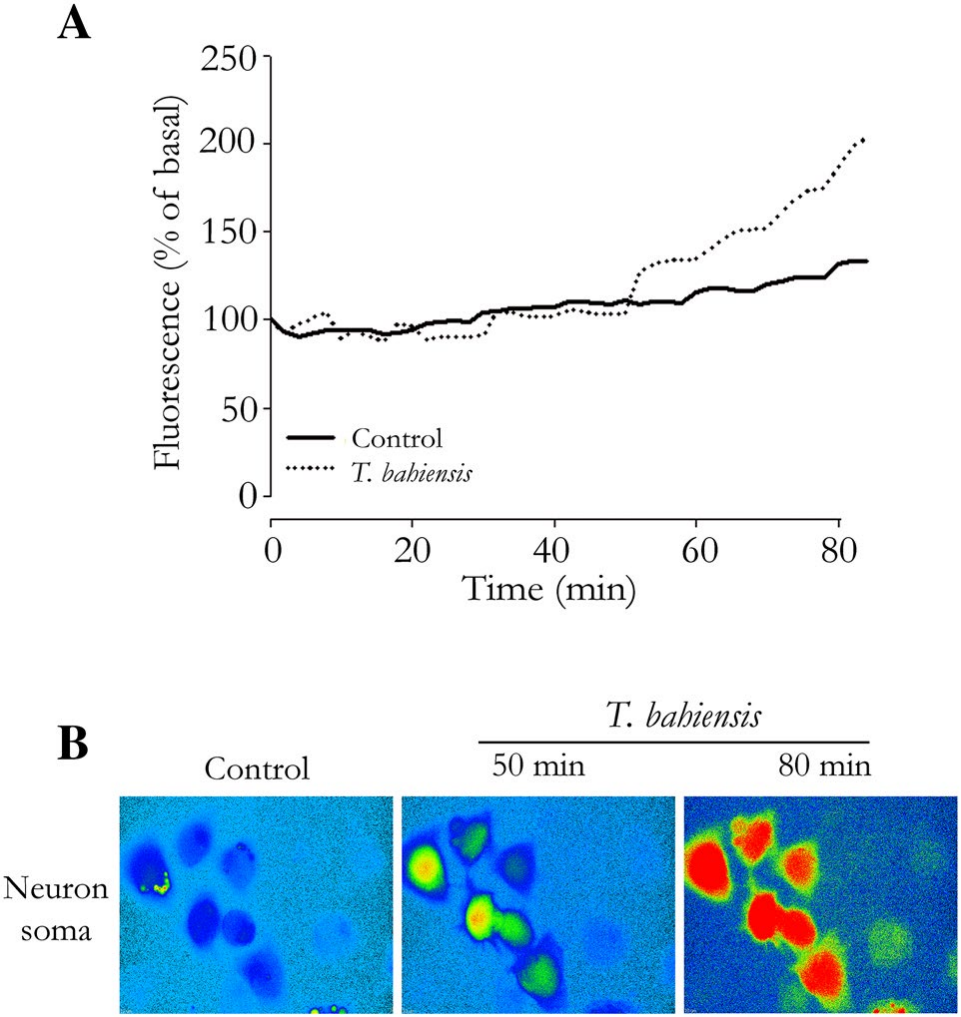
Calcium mobilization in ND7-23*wt* neurons incubated with *T. bahiensis* venom (0.3 µg/ml). Calcium movement was monitored in cells loaded with the Ca^2+^-sensitive fluorescent dye Fluo-4AM. Note the time-dependent increase in calcium mobilization from 50 min onwards. The lines in (**a**) and the cells in (**b**) are representative recordings from 11–22 cells in 5–7 experiments. Fluorescence scale (from low to high calcium concentration): light blue < dark blue < green < yellow < red. Magnification in **b**: 100 ×

Intracellular calcium movement was assessed by loading ND7-23*wt* neurons with the Ca^2+^ fluorescent marker Fluo-4-AM. Control assays showed that the neurons could be kept under the experimental conditions for 80 min with no important changes in their fluorescence or their responsiveness to depolarization by KCl. Incubation with *T. bahiensis* venom (0.3 µg/mL) resulted in a time-dependent increase in fluorescence from 50 min of incubation onwards (Fig. 8). In the first 5 min after venom addition, there were several small, rapid changes in fluorescence that became less frequent and disappeared over the time course of the experiment (data not shown).

## Discussion

*Tityus* sp. scorpion venoms are well-known in the clinic for their actions on the sympathetic and parasympathetic nervous system that account for most of the clinical manifestations of envenomation (Chippaux 2012; Bucaretchi et al. 2014; Isbister and Bawaskar 2014). A better understand of the venom’s mechanism of action is essential in order to improve envenomation therapeutics. Hence, in this study, we examined the toxicological activities of *T. bahiensis* venom on sympathetic neurotransmission using the vas deferens and a number of complimentary techniques, i.e., myography, electrophysiology and calcium imaging.

We found that in both epididymal and prostatic ends of vas deferens, a low concentration of *T. bahiensis* venom facilitated nerve-evoked contractions, while a higher venom concentration lead to total blockade of the contraction. The venom also induced an immediate contracture of both portions of rat vas deferens and, since the smooth muscle voltage-gated inward current is mainly driven by Ca^2+^ channels and the TTX-sensitive voltage-gated sodium channels are key to sympathetic neuronal action potentials generation, the use of TTX can be useful to inhibit the generation of electric impulses and exclude a presynaptic site of (but not a postsynaptic) action. Hence, the venom-induced sustained contraction was abolished TTX, confirming a pre-synaptic/nerve-dependent mechanism of action. Similar activity was also observed previously at skeletal neuromuscular junctions (Collaço et al. 2019).

Moreover, the pre-incubation of both vas deferens portions with prazosin (a non-selective α_1_-adrenoceptor antagonist) partially reduced the venom-induced contraction but only in the epididymal end while PPADS (P2X receptor antagonist) abolished this activity in both portions of the vas deferens. These findings indicate that the venom-induced sustained contraction is due to neurogenic release of both ATP and NA, with ATP being the primary mediator involved. Contractions of the vas deferens can be modulated by acetylcholine (Wallace et al. 2015) and that prejunctional nicotinic receptor activation can lead to ATP release from the nerve terminals (Williams et al. 2011), however, our experiments with *d*-Tc strongly suggests that the venom-induced sustained contractions exclude the involvement of presynaptic nicotinic receptor activation.

In addition to the sustained contraction, *T. bahiensis* venom induced several small intermittent spikes, which were faster and at a higher frequency in the epididymal end, and slower and less frequent on the prostatic end. These spontaneous contractions were abolished by TTX and markedly reduced by prazosin. Since intermittent spikes have been associated with calcium mobilization following the α_1_-adrenoreceptor activation (Tambaro S 2005), our results suggest that these venom-induced small spontaneous contractions are due to release of NA and the subsequent activation of adrenergic receptor. It worth mentioning that, in the epididymal end (where noradrenaline is the dominant neurotransmitter), the pre-incubation with PPADS markedly increased these spontaneous contractions; however, the pharmacology involved seems more complex and additional studies are required to elucidate this interaction.

Concentration–response curves to exogenous NA and ATP in presence of venom were also performed in both portions of the vas deferens to determine if the venom interacts with postsynaptic receptors that could account for facilitation and/or blockade of contractile responses. We have demonstrated that, after blocking the neuronal activity by TTX, the venom did not affect the responses to ATP and NA, confirming one more time the presynaptic origin of *T. bahiensis* effects on sympathetic transmission.

As the spontaneous release of ATP was the primary mediator responsible for the venom-induced sustained contraction, we designed an electrophysiological study to determine the effect of the venom on spontaneous excitatory junction potentials (SEJP), which reflect the smooth muscle membrane depolarization promoted by the spontaneous release of ATP via interaction with postsynaptic P2X_1_ purinoceptors; the experiments were performed in the prostatic portion only, as the amount of ATP released by epididymal end is insufficient to produce measurable SEJP (Knight et al. 2003). *T. bahiensis* venom markedly increased SEJP amplitude and frequency, which occurs at the same time as the depolarization of the smooth muscle membrane potential. A previous study showed that addition of exogenous ATP to guinea pig vas deferens induces depolarization of the membrane potential, leading to muscle contraction (Wakui et al. 1987). Therefore, in our study, the venom-mediated ATP release could be responsible for the higher SEJP amplitude and frequency, as well as for the spontaneous sustained contracture. Increases in ATP release and/or SEJP frequency by TsTX-I (a Na_V_ channel toxin isolated from *T. serrulatus* venom) and *Leiurus quinquestriatus* scorpion venom were reported in rat and mouse vas deferens (Einhorn and Hamilton 1977; Conceição et al. 2005). Similar activity (increase in spontaneous neurotransmitter release coinciding with RMP depolarization and sustained contraction) is also reported in neuromuscular junction, which was attributed to the venom-induced modulation of voltage-gated sodium channels (Collaço et al. 2019).

Based on electrophysiological characteristics and their binding sites on Na_v_ channels, two classes of known long-chain scorpion toxins have been described, namely the α- and β-toxins (He et al. 2017). The α-toxins bind to segment three of the Na_v_ channel’s domain IV and stabilize the channel in its open state, thereby inhibiting fast inactivation, thus prolonging the duration of action potentials and inducing repetitive action potential (Possani et al. 1999; Bosmans and Tytgat 2007). On the other hand, the β-toxins binds to segment four of the Na_v_ channel’s domain II and trap the voltage sensor in a pre-activated state, enhancing the channel opening, allowing the channel activation at a potential closer to the RMP (Cohen et al. 2005; Campos et al. 2007; Pedraza Escalona and Possani 2013).

To evaluate the activity of *T. bahiensis* on the sodium-mediated currents, we used ND7-23 wild type cell lines which sodium-mediated currents are largely generated by Na_v_1.7 (Rogers et al. 2016), a sodium channel subtype highly expressed in the peripheral nervous system and exerts a major whole in sympathetic neurons (Rice et al. 2015; Alonso et al. 2016; Wang et al. 2017). In our study, the sodium-mediated currents in control conditions started at − 35 mV, peaked on − 5 mV and reversed at + 44.6  ±  2.3 mV, which is consistent with a previous study (Kennedy et al. 2013). After incubating with *T. bahiensis* venom, the activation and the current–voltage curves were shifted to more negative potentials (to the left) and the current-peak amplitude increased. Similar results were reported with *T. bahiensis* venom using sensory dorsal root ganglia (DRG) neurons and ventricular cardiomyocytes (Moraes et al. 2011). *T. bahiensis* venom also markedly reduced the rate of Na_v_-current inactivation in these cells (Moraes et al. 2011). Next, we evaluated the activity of *T. bahiensis* on the spontaneous movement of intracellular Ca^2+^ in ND7-23 neuron soma. The venom induced spikes in fluorescence immediately after its addition and the fluorescence continued to a progressive increase over the duration of the experiment. Since the venom did not cause any cellular damage, it is most likely that the increased intracellular Ca^2+^ reflected partial depolarization of the nerve terminal membrane rather than venom-induced cellular death. Taken together the data would suggest that these changes are the consequence of Na_V_-channel opening resulting in membrane depolarisation and the resulting in the opening of voltage-gated Ca^2+^ channels and/or Ca^2+^ release from internal storage (Vetter and Lewis 2010; Grienberger and Konnerth 2012).

In summary, our results show that *T. bahiensis* venom at a low concentration (which better reflects a human envenomation situation) promotes a facilitatory effect on nerve-evoked vas deferens contractions by a presynaptic mechanism of action, there is no evidence for direct activation of postsynaptic receptors or damaging the smooth muscle cells. At high venom concentrations (which better reflects a scorpion sting in a smaller prey), the nerve-evoked contractions were blocked by venom. The venom also promoted a transient TTX-sensitive sustained contraction and resting membrane depolarization mediated mainly by a massive ATP release that is likely the consequence of depolarization of nerve terminal. In a manner analogous to our previous findings for the action of this venom in somatic neurotransmission (Collaço et al. 2019), we conclude that *T. bahiensis* venom prolongs axonal and nerve terminal repolarization mainly by primarily by modulating the activity of neuronal Na_V_ channels. This prolongation of repolarization enhances neurotransmitter release and facilitates nerve-evoked muscle contraction. This data shows the importance of targeting Na_V_ channel toxins when developing a therapeutic strategy to alleviate the physiological changes following being envenomed by *T. bahiensis*.

## Abbreviations

ATP: Adenosine 5′-triphosphate
*d*-Tc: *d*-Tubocurarine
EFS: Electric field stimulation
NA: Noradrenaline
Na_v_: Voltage-gated sodium (channel)
PPADS: Pyridoxalphosphate-6-azophenyl-2′,4′-disulfonic acid
RMP: Resting membrane potential
SEJP: Spontaneous excitatory junctional potential
TTX: Tetrodotoxin
